# Using time-varying models to estimate post-transplant survival in pediatric liver transplant recipients

**DOI:** 10.1371/journal.pone.0198132

**Published:** 2018-05-31

**Authors:** Cindy L. Bryce, Chung Chou H. Chang, Yi Ren, Jonathan Yabes, Gabriel Zenarosa, Aditya Iyer, Heather Tomko, Robert H. Squires, Mark S. Roberts

**Affiliations:** 1 Department of Health Policy and Management, Graduate School of Public Health, University of Pittsburgh, Pittsburgh, Pennsylvania, United States of America; 2 Department of Medicine, School of Medicine, University of Pittsburgh, Pittsburgh, Pennsylvania, United States of America; 3 Department of Clinical and Translational Science, School of Medicine, University of Pittsburgh, Pittsburgh, Pennsylvania, United States of America; 4 Department of Biostatistics, Graduate School of Public Health, University of Pittsburgh, Pittsburgh, Pennsylvania, United States of America; 5 Department of Industrial Engineering, Swanson School of Engineering, University of Pittsburgh, Pittsburgh, Pennsylvania, United States of America; 6 Department of Pediatrics, School of Medicine; Children’s Hospital of Pittsburgh, University of Pittsburgh, Pittsburgh, Pennsylvania, United States of America; Texas A&M University, UNITED STATES

## Abstract

**Purpose:**

To distinguish clinical factors that have time-varying (as opposed to constant) impact upon patient and graft survival among pediatric liver transplant recipients.

**Methods:**

Using national data from 2002 through 2013, we examined potential clinical and demographic covariates using Gray’s piecewise constant time-varying coefficients (TVC) models. For both patient and graft survival, we estimated univariable and multivariable Gray’s TVC, retaining significant covariates based on backward selection. We then estimated the same specification using traditional Cox proportional hazards (PH) models and compared our findings.

**Results:**

For patient survival, covariates included recipient diagnosis, age, race/ethnicity, ventilator support, encephalopathy, creatinine levels, use of living donor, and donor age. Only the effects of recipient diagnosis and donor age were constant; effects of other covariates varied over time. We retained identical covariates in the graft survival model but found several differences in their impact.

**Conclusion:**

The flexibility afforded by Gray’s TVC estimation methods identify several covariates that do not satisfy constant proportionality assumptions of the Cox PH model. Incorporating better survival estimates is critical for improving risk prediction tools used by the transplant community to inform organ allocation decisions.

## Introduction

Liver allocation and transplantation policies in the US must balance the problems of donor organ scarcity with mandates for fair and effective allocation. In response, oversight of the allocation process has evolved to include development and maintenance of national registry data for patients who are listed and/or transplanted [1]; development of biologically-based models of disease progression for end-stage organ failure [2]; and use of simulation models that incorporate registry data with clinical data regarding disease progression in the absence of transplantation [3]. Simulation models for adults have been critical in providing timely, evidence-based data to the transplant community. They inform policy development and evaluate the potential impact of changes before policy adoption or implementation, by comparing survival and quality of life (with and without transplantation) under different recommendations and practices [3].

The Pediatric Acute Liver Failure (PALF) study funded by the National Institute of Diabetes and Digestive and Kidney Diseases (NIDDK) is a multicenter study focused on children diagnosed with PALF. Diagnoses associated with PALF differ significantly from those seen in adults; in particular, specific diagnoses are not established in up to 50% of pediatric cases [4]. Reliable models to predict outcomes with the native liver in PALF are not yet available. Previous attempts, including Kings College Criteria [5] and the Liver Injury Unit score [6], do not reliably predict death [7, 8], but recent modeling efforts to predict outcomes in PALF have shown promise. A unique immune and inflammatory cytokine network associated with death has been identified, while an entirely different network occurred in subjects who survived with their native liver [9]. Additionally, serial assessments of clinical parameters (international normalized ratio (INR), total bilirubin, and clinical encephalopathy) differentiated subject outcomes using a growth mixture model [10]. These data inform natural history of PALF and could be used in developing predictive models for liver transplant decisions.

As collaborators with the PALF study, we are expanding prior work with a simulation model of liver transplant candidates originally calibrated for adults only [2]. Extending the model to include children improves outcome prediction for all recipients and better represents the competition for resources that are not confined within diagnosis, clinical condition, or age. The complexities of optimizing liver transplant decisions in PALF cannot be underestimated. Clinical components must be integrated, such as predicting clinical deterioration associated with death or permanent organ damage; assessing the impact of allocation decisions for both acute and chronic conditions; and estimating quality outcomes following transplantation.

Integral to developing a liver transplant decision model for PALF, the focus of this report is to demonstrate optimal methods for predicting post-transplant outcomes in pediatric transplant recipients, including children with PALF, by comparing conventional estimation methods with those that are more flexible but also more challenging to employ.

Separate but analogous analyses were conducted for two primary outcomes—post-transplant patient survival and graft survival—and survival curves were generated to illustrate effects over time for transplant recipients with a representative set of covariates. The University of Pittsburgh Institutional Review Board approved this study.

## Methods and materials

### Methods

Since February 2002, risk of death *without transplantation* for liver transplant candidates has been estimated using severity of illness calculations, specifically Pediatric End-Stage Liver Disease (PELD) scores for children less than 12 years old [11] and Model for End-Stage Liver Disease (MELD) for adults and children over 12 years old [12]. MELD/PELD calculations are widely accepted for predicting pre-transplant deaths, but there is no consensus for using MELD/PELD calculations to predict death *after* transplantation. Researchers are divided, with some studies reporting associations between pre-transplant MELD scores and post-transplant survival[13, 14] and others finding no significance [15, 16]. In one systematic review, most of the included studies found moderate associations, but overall findings indicated low levels of evidence for using MELD to estimate post-transplant survival [17].

Instead, post-transplant outcomes rely on survival models, including our earlier work [18, 19]. The Cox proportional hazards (PH) model is most commonly used [20], not only in the context of organ transplantation but for myriad health conditions and time-to-event analyses. The Cox PH model is convenient because of widespread availability in statistical packages, but it imposes several strict assumptions, namely that the effect of a variable is both proportional and constant over all future time. When these assumptions are not satisfied, Cox PH models are inappropriate. For example, in adults listed for liver transplantation, use of life support at time of transplantation has an immediate effect on the likelihood of survival but later becomes irrelevant, while prior cytomegalovirus (CMV) infection has minimal impact initially but becomes increasingly important over time [2]; proportionality assumptions are also violated in children diagnosed with hepatocellular carcinoma [18]. In these circumstances, flexible models allowing for covariate effects to change over time would improve the accuracy of survival predictions and also provide insights for better outcomes through pre-transplant management.

Gray’s piecewise-constant time-varying coefficients (TVC) model [21] is one such alternative that first tests the proportional hazards assumption and then, if not satisfied, allows but does not force the effect of a covariate (e.g., ventilator support) to vary over time. In essence, Gray’s TVC model treats the covariate effect as a *series* of Cox PH models—as the name suggests, it is “piecewise constant” within a given time interval that may be adjusted up or down in subsequent intervals. Gray’s TVC model captures important changes in covariate effects and is an extension of Cox, but estimation is more complicated and not easily available in software.

### Data sources

We obtained a standard de-identified data file of transplant candidates from United Network for Organ Sharing (UNOS), based on Organ Procurement and Transplantation Network (OPTN) data. The main file contains patient diagnoses (Table 1) and demographics for everyone placed on the transplant waiting list; it measures clinical characteristics at time of listing and (if applicable) time of transplantation. The file also maintains updated information about survival and liver function until candidates die, are removed from the waiting list, or receive a transplant. Changes in physiology are recorded using laboratory values affecting the candidate’s MELD score (albumin, bilirubin, INR, and serum creatinine) or PELD score (albumin, bilirubin, INR, age, and growth failure), plus other comorbidities and laboratory values related to severity (ascites, encephalopathy, serum sodium).

**Table 1:** Mapping of primary diagnosis in OPTN/UNOS to liver disease categories.

Main categories	Detailed categories	UNOS description
Acute liver failure	Acute liver failure	Ahn: Etiology Unknown; Ahn: Other Drug Specify; Ahn: Type A; Ahn: Type B- Hbsag+; Ahn: Non A- Non B; Ahn: Type C; Ahn: Type D; Ahn: Type B And C; Ahn: Type B And D; Ahn: Other, Specify (E.G., Acute Viral Infection, Autoimmune Hepatitis–Fulminant)
Autoimmune disorder	Autoimmune, cryptogenic	Cirrhosis: Drug/Indust Exposure Other Specify; Cirrhosis: Cryptogenic-Idiopathic; Cirrhosis: Other, Specify (E.G., Histiocytosis, Sarcoidosis, Granulamatous); Cirrhosis: Autoimmune; Cirrhosis: Cryptogenic (Idiopathic)
Cancer	Cancer	Plm: Hepatoma–Hepatocellular Carcinoma; Plm: Hepatoma (Hcc) And Cirrhosis, Plm: Fibrolamellar (Fl-Hc); Plm: Cholangiocarcinoma (Ch-Ca); Plm: Hepatoblastoma (Hbl); Plm: Hemangioendothelioma, Hemangiosarcoma, Angiosarcoma; Plm: Other Specify (I.E., Klatzkin Tumor, Leiomysarcoma); Bile Duct Cancer: (Cholangioma, Biliary Tract Carcinoma); Secondary Hepatic Malignancy Other Specify
Metabolic disease	Metabolic disorder	Metdis: Alpha-1-Antitrypsin Defic A-1-A; Metdis: Wilson’s Disease, Other Copper Metabolism Disorder; Metdis: Hemochromatosis-Hemosiderosis; Metdis: Glyc Stor Dis Type I (Gsd-1); Metdis: Glyc Stor Dis Type Ii (Gsd-Iv); Metdis: Hyperlipidemia-Ii, Homozygous Hypercholesterolemia; Metdis: Tyrosinemia; Metdis: Primary Oxalosis/Oxaluria, Hyperoxalauria; Metdis: Maple Syrup Urine Disease; Metdis: Other Specify
Other chronic disease	Primary biliary cirrhosis	Primary Biliary Cirrhosis (Pbc)
	Primary sclerosing cholangitis	Psc: Crohn’s Disease; Psc: Ulcerative Colitis; Psc: No Bowel Disease; Psc: Other Specify
	Hepatitis C virus	Cirrhosis: Type Non A, Non B; Cirrhosis: Type C; Cirrhosis: Type D; Cirrhosis: Type B And C; Cirrhosis: Type B And D; Alcoholic Cirrhosis With Hepatitis C;
	Hepatitis B virus	Cirrhosis: Type B- Hbsag+
	Miscellaneous, other	Cirrhosis: Type A; Cirrhosis: Chronic Active Hepatitis: Etiology Unknown; Cirrhosis: Fatty Liver (Nash); Acute Alcoholic Hepatitis; Neonatal Hepatitis Other Specify; Congenital Hepatic Fibrosis; Cystic Fibrosis; Benign Tumor: Hepatic Adenoma; Benign Tumor: Polycystic Liver Disease; Benign Tumor: Other Specify; Graft Vs. Host Dis Sec To Non-Li Tx; Trauma Other Specify; Hepatitis B: Chronic Or Acute; Hepatitis C: Chronic Or Acute; Na: Non-Hd Follow-ups Only; Budd-Chiari Syndrome
Biliary atresia	Biliary atresia/hypoplasia	Sec Biliary Cirrhosis: Caroli’s Disease; Sec Biliary Cirrhosis: Choledochol Cyst; Sec Biliary Cirrhosis: Other Specify; Familial Cholestasis: Byler’s Disease; Familial Cholestasis: Other Specify; Choles Liver Disease: Other Specify; Neonatal Cholestatic Liver Disease; Biliary Atresia: Extrahepatic; Biliary Hypoplasia: Nonsyndromic Paucity Of Intrahepatic Bile Duct; Biliary Hypoplasia: Alagille’s Syndrome (Paucity Of Intrahepatic Bile Duct); Tpn/Hyperalimentation Ind Liver Disease

For transplant recipients, the database additionally provides demographic and clinical information about the donor organ; post-transplant follow-up information and lab values; and re-listing and re-transplantation information, if applicable.

### Outcome variables and model covariates

We considered two outcome variables, *post-transplant patient survival* (number of days between primary liver transplant and death) and *graft survival* (number of days between primary liver transplant and death or re-transplantation).

We identified potential covariates based on prior work [18, 19] and expertise of the PALF oversight committee. MELD/PELD score was excluded from consideration because, as noted above, it was developed for estimating survival *without* transplantation. For both outcomes, we considered the same initial covariates related to the recipient, the donated organ, and the transplant procedure (Tables 2 and 3).

**Table 2:** Characteristics of pediatric liver transplant recipients during the MELD/PELD era*.

Covariates		All recipients (N = 3,175)	Subgroups (stratified by posttransplant outcome)		
			Alive (N = 2,566)	Died (N = 390)	Retransplanted (N = 219)
**Demographics**					
Age in years, median, mean ± SD		1.0, 4.8 ± 5.9	1.0, 4.7 ± 5.8	1.0, 5.3 ± 6.4	1.0, 5.1 ± 6.3
Age group, No. (%)					
	< 12 years	2,549 (80.3)	2,085 (81.3)	290 (74.4)	174 (79.5)
	≥ 12 years	626 (19.7)	481 (18.7)	100 (25.6)	45 (20.5)
Gender, No. (%)					
	Female	1,627 (51.2)	1,306 (50.9)	207 (53.1)	114 (52.0)
	Male	1,548 (48.8)	1,260 (49.1)	183 (46.9)	105 (48.0)
Race / ethnicity, No. (%)					
	White	1,627 (51.2)	1,317 (51.3)	184 (47.2)	126 (57.5)
	Black	542 (17.1)	419 (16.3)	87 (22.3)	36 (16.4)
	Hispanic	723 (22.8)	592 (23.1)	91 (23.3)	40 (18.3)
	Asian	175 (5.5)	152 (5.9)	16 (4.1)	7 (3.2)
	Other	108 (3.4)	86 (3.4)	12 (3.1)	10 (4.6)
Primary source of payment, No. (%)					
	Private insurance	1,548 (48.8)	1,266 (49.3)	168 (43.1)	114 (52.1)
	Public Insurance	1,413 (44.5)	1,120 (43.6)	197 (50.5)	96 (43.8)
	Self	53 (1.7)	45 (1.8)	5 (1.3)	3 (1.4)
	Other/unknown	161 (5.1)	135 (5.3)	20 (5.1)	6 (2.7)
**Medical/Clinical covariates at time of transplantation**					
Liver disease, No. (%)					
	Biliary atresia	1,487 (46.8)	1,239 (48.3)	143 (36.7)	105 (48.0)
	Acute liver failure	478 (15.1)	366 (14.3)	88 (22.6)	24 (11.0)
	Autoimmune disease	232 (7.3)	189 (7.4)	23 (5.9)	20 (9.1)
	Metabolic disorder	422 (13.3)	340 (13.3)	50 (12.8)	32 (14.6)
	Other chronic disease	556 (17.5)	432 (16.8)	86 (22.1)	38 (17.4)
Blood type, No. (%)					
	A	1,073 (33.8)	861 (33.6)	128 (32.8)	84 (38.4)
	AB	407 (12.8)	332 (12.9)	47 (12.1)	2 (0.9)
	B	129 (4.1)	112 (4.4)	15 (3.8)	28 (12.8)
	O	1,566 (49.3)	1,261 (49.1)	200 (51.3)	105 (48.0)
On ventilator, No. (%)					
	Yes	332 (10.5)	222 (8.65)	84 (21.5)	26 (11.9)
	No	2,843 (89.5)	2,344 (91.3)	206 (78.5)	193 (88.1)
Laboratory values, Median, Mean ± SD ^§^					
	Albumin (g/dl)	3.1, 3.1 ± 0.9	3.1, 3.1 0.8	3.0, 3.1 ± 0.7	3.0, 3.1 ± 0.7
	Total bilirubin (mg/dl)	8.8, 11.8 ± 11.2	8.6, 11.6 ± 11.1	11.1, 13.3 ± 12.0	7.6, 11.2 ± 11.3
	Serum creatinine (mg/dl)^║^	0.3, 0.5 ± 0.5	0.3, 0.5 ± 0.5	0.4, 0.6 ± 0.9	0.3, 0.5 ± 0.5
	INR	1.5, 2.1 ± 2.5	1.5, 2.1 ± 2.7	1.7, 2.4 ± 2.0	1.5, 2.0 ± 1.4
	Sodium^§^	138.0, 138.2 ± 5.5	138.0, 138.1 ± 5.3	138.0, 139.0 ± 6.7	138.0, 138.3 ± 4.6
Growth failure, No. (%)^¶^					
	Yes	834 (26.3)	667 (26.0)	114 (29.2)	53 (24.2)
	No	1,987 (62.6)	1,592 (62.0)	243 (62.3)	152 (69.4)
	Unknown	354 (11.1)	307 (12.0)	33 (8.5)	14 (6.4)
Presence of ascites, No. (%)					
	Yes	1,366 (43.0)	1,077 (42.0)	187 (47.9)	102 (46.5)
	No	1,091 (34.4)	871 (33.9)	135 (34.6)	85 (38.3)
	Unknown	718 (22.6)	618 (24.1)	68 (17.4)	32 (14.6)
Presence of encephalopathy, No. (%)					
	Yes	282 (8.9)	190 (7.4)	69 (17.7)	23 (10.5)
	No	2,773 (87.3)	2,283 (89.0)	299 (76.7)	191 (87.2)
	Unknown	120 (3.8)	93 (3.62)	22 (5.6)	5 (2.3)
CMV status, No. (%)					
	Yes	1,133 (35.7)	911 (35.5)	156 (40.0)	66 (30.1)
	No	1,997 (62.9)	1,616 (63.0)	233 (59.7)	148 (67.6)
	Missing	45 (1.4)	39 (1.5)	1 (0.3)	5 (2.3)
EBV status, No. (%)					
	Yes	1,114 (35.1)	890 (34.7)	147 (37.7)	77 (35.2)
	No	2,016 (63.5)	1,637 (63.8)	242 (62.1)	137 (62.6)
	Missing	45 (1.4)	39 (1.5)	1 (0.3)	5 (2.3)
**Other covariates (not included in analyses)**					
Scoring system used, No. (%)					
	PELD	2,736 (86.2)	2,216 (86.4)	331 (84.9)	189 (86.3)
	MELD	439 (13.8)	350 (13.6)	59 (15.1)	30 (13.7)
Calculated score at time of transplant, median, mean ± SD					
	PELD score	16.0, 16.2 ± 14.3	16.0, 15.9 ± 14.3	19.0, 19.1 ± 14.2	16.0, 15.5 ± 13.7
	MELD score	20.0, 22.4 ± 11.1	19.0, 21.7 ± 10.9	26.0, 26.3 ± 11.6	20.5, 23.4 ± 11.0
Status 1 at time of transplant, No. (%)					
	Yes	977 (30.8)	743 (29.0)	168 (43.1)	66 (30.1)
	No	2198 (69.2)	1823 (71.0)	222 (56.9)	153 (69.9)
Active exception at time of transplant, No. (%)					
	Yes	730 (23.0)	606 (23.6)	74 (19.0)	50 (22.8)
	No	2,445 (77.0)	1,960 (76.4)	316 (81.0)	169 (77.2)
Waiting time (days), median, mean ± SD		45.0, 109.3 ± 188.0	48.0, 114.0 ± 191.3	27.0, 77.8 ± 145.8	44.0, 110.7 ± 210.2

* Because the observation period is not fixed and outcomes for some recipients (alive, retransplanted) are related to length of follow-up, the characteristics in Table 2 are purely descriptive; hence, p-values are not provided.

^║^ Serum creatinine values were missing for 109 children (89 alive, 5 retransplanted, and 15 dead).

^§^ Sodium values were missing for 883 children (666 alive, 78 retransplanted, and 139 dead).

^¶^ Growth failure is measured at time of listing, based on height and weight ranges (adjusted for age and sex) [7].

**Table 3:** Donor- and transplantation-related characteristics for patient cohort.

Covariates		All recipients	Subgroups (stratified by posttransplant outcome)		
		(N = 3,175)	Alive (N = 2,566)	Died (N = 390)	Retransplanted (N = 219)
**Donor-related**					
Type of donor, No. (%)					
	Deceased	2,755 (86.8)	2,207 (86.0)	352 (90.3)	196 (89.5)
	Living	420 (13.2)	359 (14.0)	38 (9.7)	23 (10.5)
Donor age in years, median, mean ± SD ^††^		14.0, 15.5 ± 14.4	13.0, 15.0 ± 13.8	13.0, 15.0 ± 13.7	13.0, 16.7 ± 17.1
Donor gender, No. (%)					
	Female	1,417 (44.6)	1,145 (44.6)	166 (42.6)	106 (48.4)
	Male	1,758 (55.4)	1,421 (55.4)	224 (57.4)	113 (51.6)
Donor race / ethnicity, No. (%)					
	White	1,902 (59.9)	1,525 (59.4)	246 (63.1)	131 (59.8)
	Black	513 (16.2)	408 (15.9)	59 (15.1)	46 (21.0)
	Hispanic	636 (20.0)	532 (20.7)	69 (17.7)	35 (16.0)
	Asian	67 (2.1)	55 (2.1)	9 (2.3)	3 (1.4)
	Other	57 (1.8)	46 (1.8)	7 (1.8)	4 (1.8)
Donor blood type, No. (%)					
	A	978 (30.8)	801 (32.1)	108 (27.7)	69 (31.5)
	AB	303 (9.5)	243 (9.5)	5 (1.3)	0 (0.0)
	B	51 (1.6)	46 (1.8)	35 (9.0)	25 (11.4)
	O	1,843 (58.0)	1,476 (57.5)	242 (62.1)	125 (57.1)
Blood type compatibility, No. (%)					
	Yes	3,071 (96.7)	2,387 (96.9)	371 (95.1)	213 (97.3)
	No	104 (3.3)	79 (3.1)	19 (4.9)	6 (2.7)
Donor CMV status, No. (%)					
	Yes	154 (5.9)	133 (5.2)	16 (4.1)	5 (2.3)
	No	2,97 (93.7)	2,394 (93.9)	373 (95.6)	209 (95.4)
	Missing	45 (1.4)	39 (1.5)	1 (0.3)	5 (2.3)
Donor EBV status, No. (%)					
	Yes	1,114 (35.1)	890 (35.7)	147 (37.7)	77 (35.2)
	No	2,016 (63.5)	1,637 (63.8)	242 (62.1)	137 (62.6)
	Missing	45 (1.4)	39 (1.5)	1 (0.3)	5 (2.3)
**Transplantation-related**					
Transplant year, No. (%)					
	2002	202 (6.4)	144 (5.6)	41 (10.5)	17 (7.8)
	2003	352 (11.1)	262 (10.2)	56 (14.4)	34 (15.5)
	2004	373 (11.7)	288 (11.2)	54 (13.8)	31 (14.2)
	2005	385 (12.1)	301 (11.7)	47 (12.1)	37 (16.9)
	2006	402 (12.7)	321 (12.5)	59 (15.1)	22 (10.1)
	2007	420 (13.2)	354 (13.8)	44 (11.3)	22 (10.1)
	2008	421 (13.3)	362 (14.1)	37 (9.5)	22 (10.1)
	2009	414 (13.0)	352 (13.7)	36 (9.2)	26 (11.9)
	2010	206 (6.5)	182 (7.1)	16 (4.1)	8 (3.7)
Transplant center region, No. (%)					
	1: CT, ME, MA, NH, RI	97 (3.1)	86 (3.4)	10 (2.6)	1 (0.5)
	2: DC, DE, MD, NJ, PA, WV	399 (12.6)	334 (13.0)	38 (9.7)	27 (12.3)
	3: AL, AR, FL, GA, LA, MS, PR	413 (13.0)	326 (12.7)	53 (13.6)	34 (15.3)
	4: OK, TX	322 (10.1)	247 (9.6)	51 (13.1)	24 (11.0)
	5: AZ, CA, NV, NM, UT	609 (19.2)	519 (20.2)	54 (13.9)	36 (16.4)
	6: AK, HI, ID, MT, OR, WA	73 (2.3)	62 (2.4)	6 (1.5)	5 (2.3)
	7: IL, MN, ND, SD, WI	270 (8.5)	211 (8.2)	35 (9.0)	24 (11.0)
	8: CO, IA, KS, MO, NE, WY	275 (8.7)	213 (8.3)	39 (10.0)	23 (10.5)
	9: NY, VT	262 (8.3)	222 (8.7)	23 (5.9)	17 (7.8)
	10: IN, MI, OH	285 (9.0)	219 (8.5)	49 (12.6)	17 (7.8)
	11: KY, NC, SC, TN, VA	170 (5.4)	127 (5.0)	32 (8.2)	11 (5.0)
Type of organ procured, No. (%)					
	Local	1,504 (47.4)	1,223 (47.7)	171 (43.8)	110 (50.2)
	Regional	1,220 (38.4)	970 (37.8)	175 (44.9)	75 (34.3)
	Other	451 (14.2)	373 (14.5)	44 (11.4)	34 (15.5)
Procurement metrics, median, mean ± SD					
	Cold ischemia time (hours)^#^	7.0, 7.1 ± 4.3	6.8, 7.0 ± 4.2	7.2, 7.7 ± 5.0	7.0, 7.1 ± 4.0
	Procurement distance (miles)**	130.0, 257.1 ± 372.0	128.0, 258.7 ± 375.8	135.5, 246.7 ± 347.0	139.0, 237.2 ± 371.3
Partial or split donor organ, No. (%)					
	Partial or split	1,867 (58.8)	1,511 (58.9)	219 (56.2)	137 (62.6)
	Whole	1,308 (41.2)	1,055 (41.1)	171 (43.8)	82 (37.4)

**Abbreviations:** IQR = interquartile range; ICU = intensive care unit.

†† The ages of five donors were missing (alive).

# Cold ischemia time values were missing for 441 children (358 alive, 42 dead, and 41 retransplanted).

** Procurement distance values were missing for four children (alive).

*Recipient characteristics*. Attributes include demographics, namely age, gender, race/ethnicity, and primary source of payment. They also include six major disease categories, aggregated in consultation with our oversight committee, that are relevant in children: biliary atresia, metabolic disorder, cancer, and autoimmune disease, plus acute liver failure and a final category for miscellaneous other chronic diagnoses (Table 1). This final group combines heterogeneous diagnoses appearing in small numbers, with “unknown” (n = 279) and primary sclerosing cholangitis (n = 66) being the most prevalent.

Other clinical covariates describe blood type, laboratory values (albumin, bilirubin, creatinine, INR, sodium), growth failure [22], ventilator support, presence of ascites or encephalopathy, and CMV and EBV status.

*Donor characteristics*. Donor-specific covariates include demographics, blood type, whether the donor was deceased or living, and CMV and EBV status.

*Transplant information*. Information describing the transplant itself included year of transplant (a proxy for secular changes), transplant center region (defined by OPTN/UNOS), a binary indicator for use of partial/split organs, cold ischemia time, and transport distance of the donor organ. Except for geographic region, information about transplant centers was not available.

### Statistical analysis

We summarized cohort characteristics using descriptive statistics, first for the entire sample and then separately for patients who remained alive (without re-transplant) for the study duration, died (without re-transplant), or were re-transplanted during the study period.

We followed the same analytical procedure for both patient and graft survival, initially testing proportionality assumptions for each covariate and estimating univariable models using Gray’s TVC model. Importantly, the Cox PH model is a special case of Gray’s; when covariates satisfy proportionality assumptions, estimates for the two models are identical. Using univariable results, we retained covariates with a *P*-value < 0.15. This subset was combined into a multivariable Gray TVC model, using a backward stepwise procedure for variable selection to gain more power.

We encountered two types of missing data: dichotomous variables (usually indicating presence or absence of a symptom/condition) that are often overlooked when absent; and values where data were not captured. In the first case, we treated missing data as “absence” of a condition and tested this in sensitivity analyses; in the second case, data were treated as truly missing and observations were dropped from the estimation.

After finalizing our specification using Gray’s TVC, we estimated the corresponding Cox PH model. The findings are summarized graphically, and we superimposed both hazard plots onto a common coordinate axis.

## Results

### Definition of patient sample

**Fig 1:**
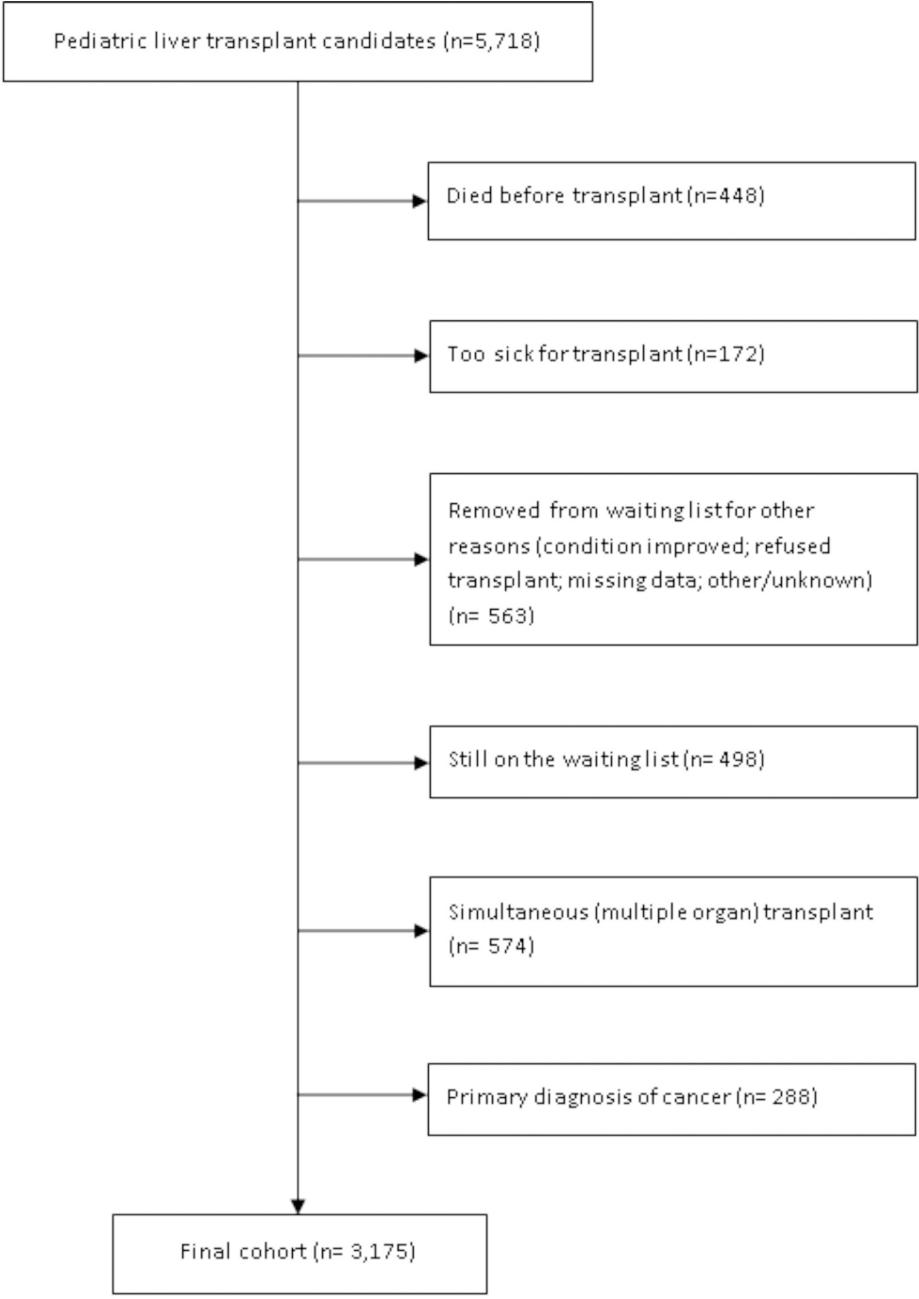
Sample cohort of pediatric transplant recipients (n = 3,175).

The OPTN data file contained 5,718 children less than 18 years old who were listed for liver transplantation between February 2002 (when MELD/PELD was implemented) and June 2010. We excluded 1,183 children who were removed from the waiting list for myriad reasons, including death (n = 448), too sick (n = 172), too healthy (n = 445), refused transplant (n = 14), missing transplantation data (n = 3), and other/unknown reasons (n = 101). We also removed children still awaiting transplantation (n = 498). Finally, we excluded 574 children receiving multiple simultaneous organ transplants (e.g., combined kidney and liver) and 288 recipients with liver cancer, who were evaluated separately [18]. The current analysis includes 3,175 pediatric liver transplant recipients initially transplanted by June 2010, with post-transplant follow-up through June 2013 (Fig 1).

### Description of patient sample

**Fig 2:**
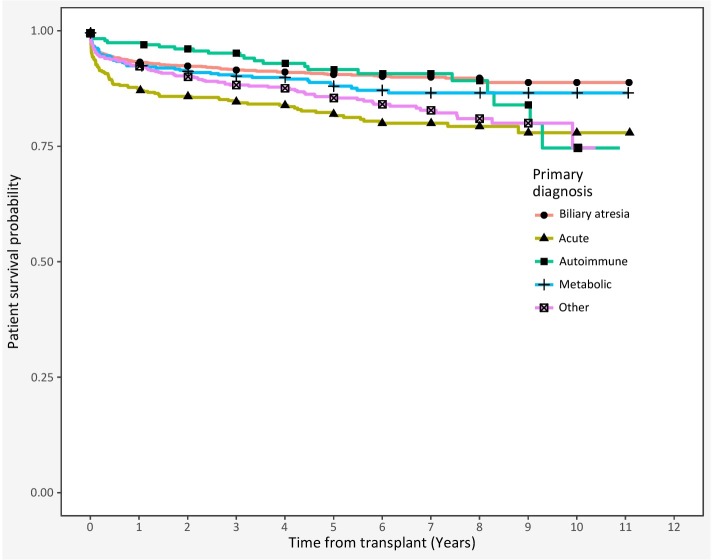
Kaplan-Meier graph of post-transplant patient survival, by liver disease category.

Transplant recipients are described in Table 2 (demographics and clinical characteristics) and Table 3 (donor- and allocation-related information). Unadjusted post-transplant survival, stratified by disease, is provided in Fig 2; in general, survival appears similar, except for recipients with acute liver failure and miscellaneous other diagnoses (logrank test, *p* < 0.001). An analogous figure for graft survival appears in supporting information (S1 Fig).

As Table 2 illustrates, most recipients remained alive throughout the study period, so this group closely resembles the overall sample. Re-transplanted patients were similar to the overall sample, although they were more likely to be white and have private insurance. Autoimmune disease and metabolic disorders were also more common, and re-transplanted recipients appeared sicker at time of (primary) transplant, with higher rates of ascites and encephalopathy.

For those who died after transplantation, there are similarities regarding gender and blood type, but they stand out from the overall sample in several respects. A higher proportion of decedents were older (at least 12 years old) at transplant, black, and publicly insured. They were more likely to have acute liver failure or miscellaneous other diagnoses (Table 1); they appeared sicker at transplant, with worse bilirubin and higher rates of ascites, CMV, encephalopathy, and ventilator support. A higher proportion was Status 1 with worse MELD/PELD scores and shorter waiting times, and proportionately fewer were active exceptions at transplant.

Factors related to the donor or procedure itself did not vary greatly, although recipients who remained alive enlisted living donors more often than those who died or were re-transplanted, and re-transplanted recipients had higher rates of partial/split donor organs for their primary transplant (Table 3). Decedents received more organs procured regionally, which reflects allocation rules prioritizing the sickest candidates (Status 1) first, followed by candidates listed in the donor’s region with highest MELD/PELD scores [23].

### Patient survival

#### Model estimation

In estimating univariable Gray’s TVC models, serum sodium was excluded because of missing values (n = 883). Of the remaining covariates, 13 were significantly associated with overall survival: recipient age, race/ethnicity, primary source of payment, liver disease, total bilirubin, creatinine, encephalopathy, ventilator support, donor age, use of a living donor, use of partial/split organs, type of organ procurement (local, regional, or national), and cold ischemia time.

Covariates were considered jointly for multivariable estimation using backward stepwise selection. The final Gray TVC model (Table 4, left-hand side) included five of the original covariates (recipient age, liver disease, creatinine, donor age, and use of living donors), plus three other covariates that were recoded and retained: race/ethnicity, which was recoded as dichotomous (black race vs. nonblack race) so that the model would converge; and a composite variable for encephalopathy and ventilator use at transplant (neither present; encephalopathy only; ventilator only; or both present), which was necessary to address collinearity between the two variables.

**Table 4:** Comparison of PATIENT survival estimates (multivariable Gray TVC vs. Cox PH models).

		Gray TVC model			Cox’s PH model		H_o_: Log hazard ratio is constant over time
		Log hazard ratio		H_o_: Log hazard ratio = 0	Log hazard ratio	Overall *p* value	
Covariate		Min	Max				
Recipient age		-0.070	0.047	< 0.001	-0.027	0.018	< 0.001
Race/ethnicity							
	Nonblack (referent)	—	—	—	—	—	—
	Black	-0.089	0.719	0.001	0.303	0.015	0.005
Diagnosis							
	Biliary atresia (referent)	—	—	—	—	—	—
	Autoimmune disease	-0.569	0.374	0.685	-0.056	0.816	n/s
	Metabolic disorder	-0.156	0.529	0.287	0.190	0.278	n/s
	Acute liver failure	-0.121	0.539	0.215	0.213	0.208	n/s
	Other chronic diagnoses	0.086	0.690	0.012	0.405	0.008	n/s
Serum creatinine at transplant		0.035	0.426	< 0.001	0.213	0.005	0.007
Encephalopathy/mechanical ventilation at transplant							
	Neither (referent)	—	—	—	—	—	—
	Ventilator only	-0.343	1.520	< 0.001	0.803	< 0.001	< 0.001
	Encephalopathy only	0.336	1.106	< 0.001	0.726	< 0.001	n/s
	Both conditions present	0.139	1.515	< 0.001	0.786	< 0.001	< 0.001
Use of living donor							
	No (referent)	—	—	—	—	—	—
	Yes	-0.939	-0.183	0.002	-0.543	0.004	0.045
Age of donor		0.005	0.021	0.001	0.012	0.002	n/s

**Fig 3:**
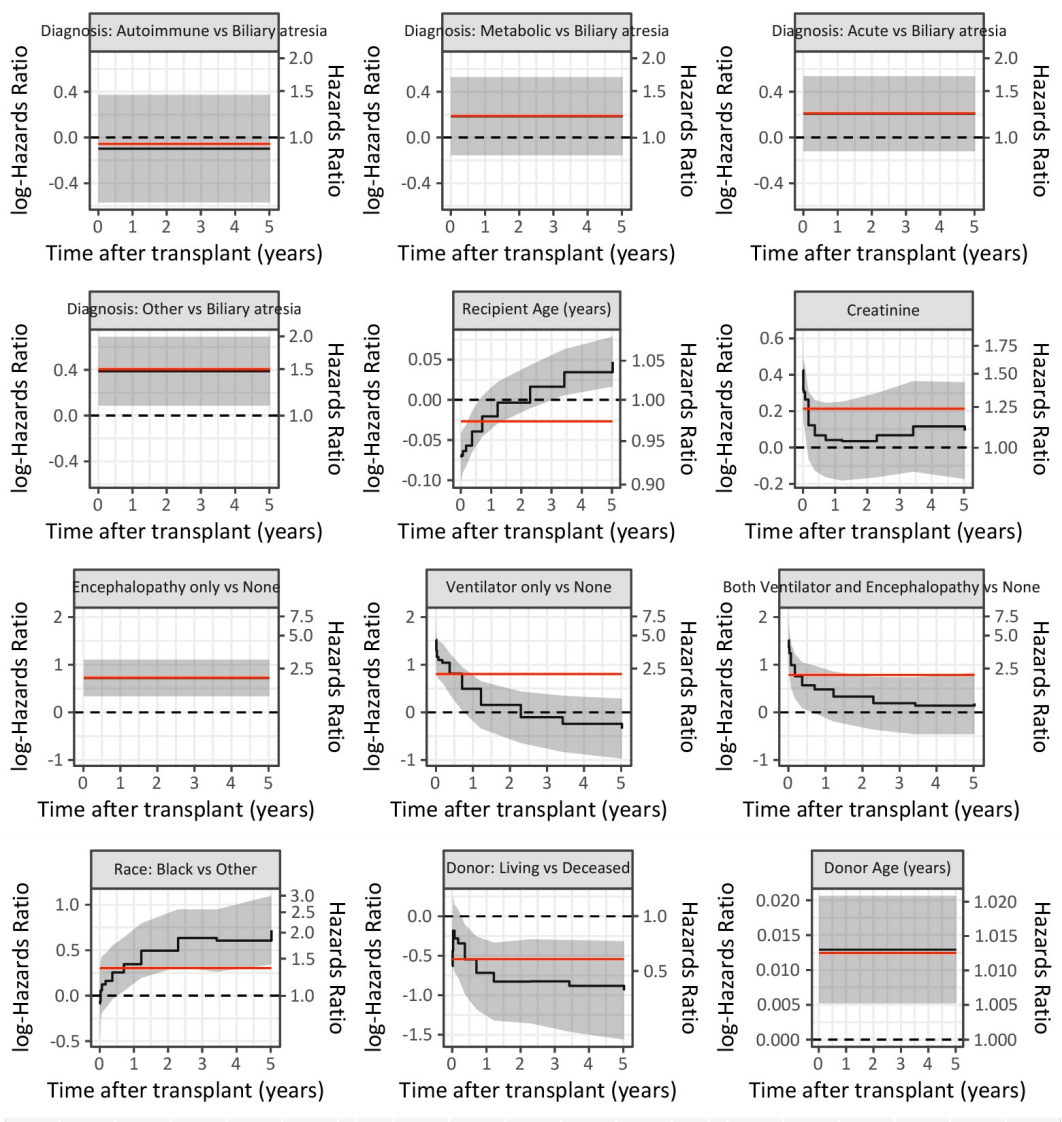
Multivariable results for patient survival. In each graph, the effect of the covariate is graphed over time based on the Cox proportional hazards model (red line) and Gray’s time-varying model (black line, with 95% confidence intervals in gray). The model estimates can be compared to a log hazard of 0 (no effect; black dashed line). Of note, when Cox’s assumptions are maintained, the Gray and Cox estimates will be the same and the red and black lines may be indistinguishable in the graph.

After estimating a Cox proportional hazards model with the same specification (Table 4, right-hand side), results for both approaches were plotted and compared (Fig 3).

#### Comparison of Gray TVC and Cox PH models

Covariate effects for both models are summarized graphically (Fig 3). For each covariate, a horizontal line at 0 (black dashed line) represents no relationship between the covariate and patient survival. Estimated hazards from the Cox PH model are constant (solid red line). Estimated log hazards and 95% confidence bands of Gray’s TVC model (solid black line, plus grey shading) are superimposed to illustrate covariate effects when hazards are not held constant.

*Liver disease*. Biliary atresia was the most common diagnosis (47%) and served as the referent category. In comparison, survival of recipients with acute liver failure, autoimmune diseases, and metabolic disorders experience was similar to that of biliary atresia (the dashed line at 0 falls in the shaded bands). Only recipients with miscellaneous other (e.g., cystic fibrosis, Budd-Chiari, neonatal hepatitis) have significantly poorer survival outcomes after transplant.

*Recipient age*. Recipient age at time of transplant has no effect initially; after 3 years, children who were older at time of transplant have a higher risk of death than those transplanted at younger ages. “Higher risk” is a relative statement: between any two age groups, older recipients have higher risk of death 3 years after transplantation. Although this statement appears self-evident, it bears mention because the cohort consists exclusively of children.

*Creatinine at transplant*. In contrast to the Cox PH model, the estimated hazards from the Gray TVC model show that elevated creatinine levels increase the recipient’s risk of post-transplant death during the first 90 days. This risk declines afterwards and later becomes nonsignificant.

*Encephalopathy/Ventilator support*. If encephalopathy is present and/or use of ventilation is required at transplant, patients experience a higher risk of death, particularly in the first 6–12 months.

*Race (black vs*. *nonblack)*. The Cox PH model indicates poorer post-transplant survival for black recipients at all time points, whereas the time-varying estimates show no differences initially but increased risk for black race over the long term.

*Use of living donors*. The Cox PH model shows immediate benefit to use of living donors. In the Gray TVC model, the initial survival benefit is not statistically significant; however, the advantages become apparent after 6 months and remain significant.

*Donor age*. Both models show that older donor organs increase risk of death and the risk is constant over time.

#### Example: Patient survival for prototypical recipient

Our findings reveal additional insights from time-varying methods, but clinical significance is harder to interpret. This section describes a pediatric transplant recipient with a typical combination of covariates (prototype) and generates survival curves to highlight covariate effects over time.

We defined a prototype using median values for continuous covariates and modal outcomes for categorical variables. Only covariates for the final model (Table 4) were considered. Hence, the recipient is 4.8 years old, non-black, with biliary atresia. At transplant, the recipient has creatinine levels of 0.48 mg/dL, has no encephalopathy, uses no ventilator support, and receives a graft from a 15.5-year old deceased donor.

**Fig 4:**
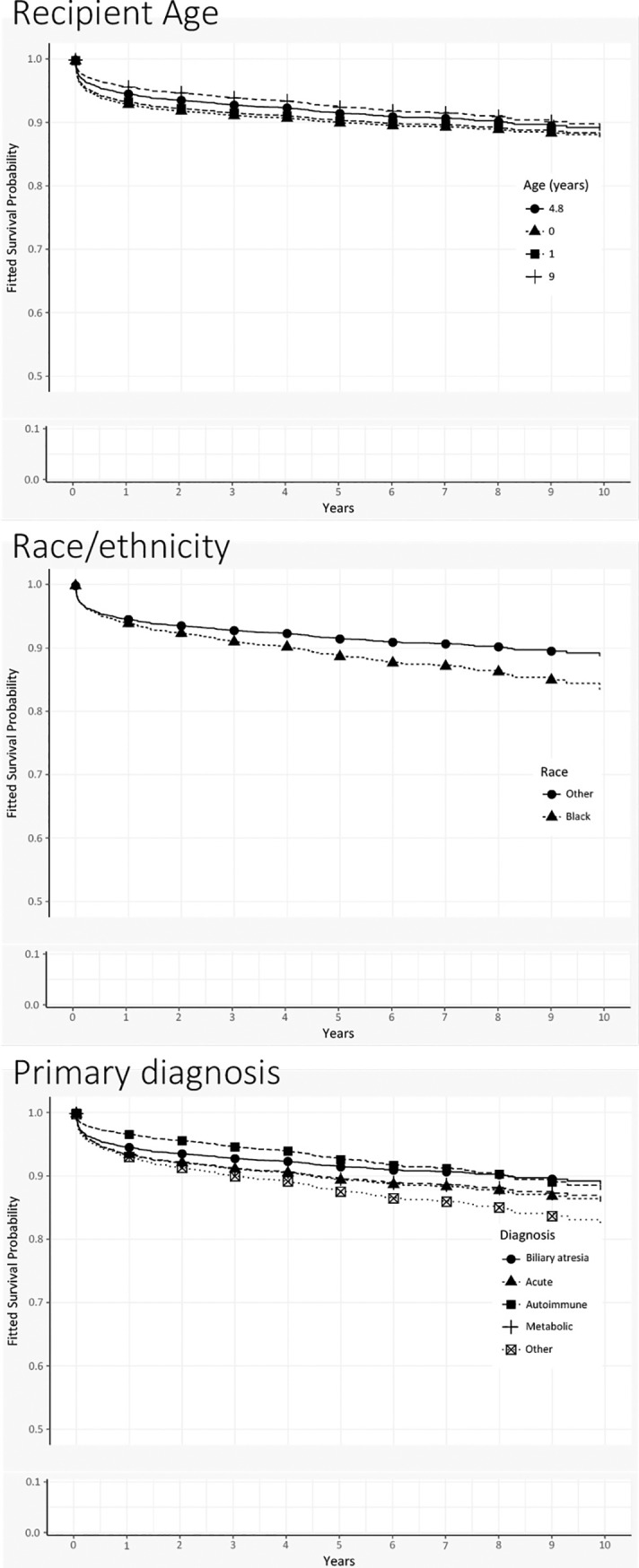
Patient survival curves for demographic covariates of Gray’s multivariable time-varying model. In each of the graphs, the “prototypical pediatric transplant recipient” uses median values for continuous variables and modal values for categorical variables. The overall survival curve for the prototype appears in each of the graphs as solid circles.

**Fig 5:**
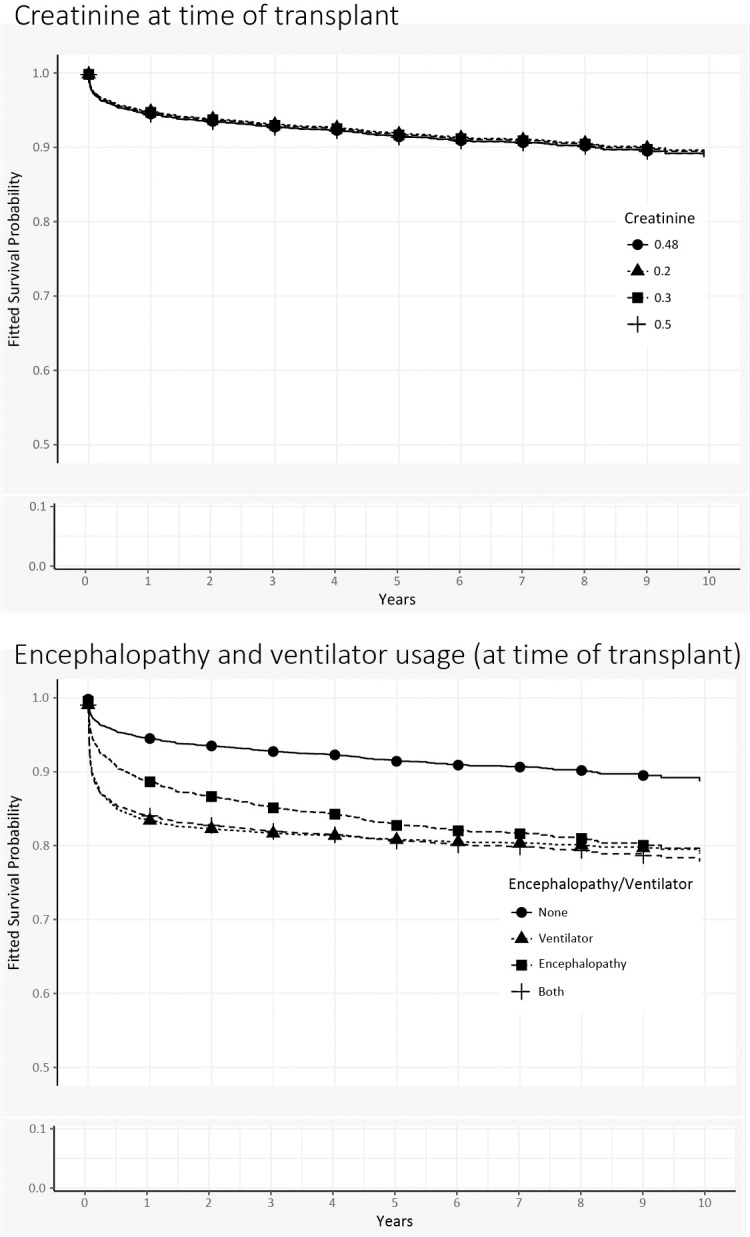
Patient survival curves for clinical covariates of Gray’s multivariable time-varying model. In each of the graphs, the “prototypical pediatric transplant recipient” uses median values for continuous variables and modal values for categorical variables. The overall survival curve for the prototype appears in each of the graphs as solid circles.

**Fig 6:**
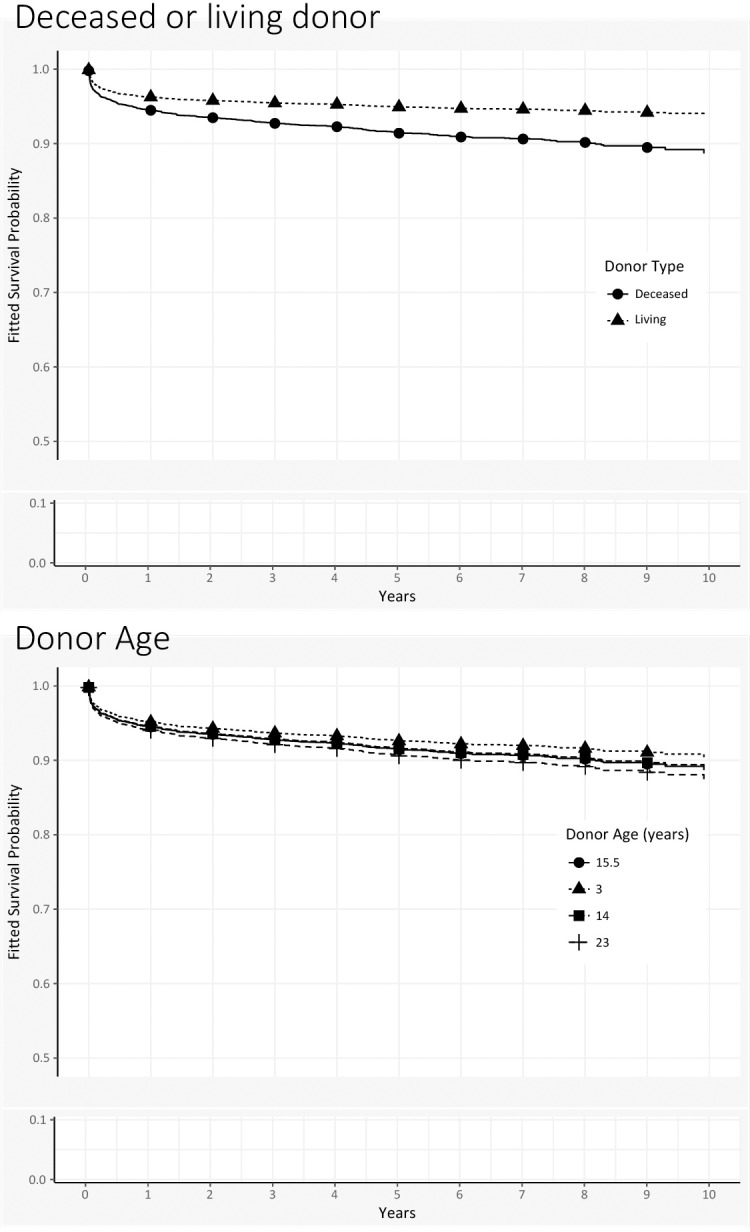
Patient survival curves for donor covariates of Gray’s multivariable time-varying model. In each of the graphs, the “prototypical pediatric transplant recipient” uses median values for continuous variables and modal values for categorical variables. The overall survival curve for the prototype appears in each of the graphs as solid circles.

We generated new survival curves and varied each covariate in turn, holding everything else at baseline values (Figs 4, 5 and 6). (Throughout the graphs, the prototype’s survival curve is the same and appears first in the legend.) For example, in the first graph (recipient age), the prototype is 4.8 years old. Among children who are older (e.g., 9 years old) at time of transplant, the survival curve lies slightly above the prototype’s, indicating better survival. Curves for younger children fall slightly below, but in general survival does not vary much by age. Moving through other graphs, we see that black recipients have worse survival than nonblack recipients. Recipients with autoimmune diseases are similar to those with biliary atresia, but other diagnoses show slightly worse survival. Although creatinine appeared significant in model estimates, survival curves at various levels of creatinine show no differences. On the other hand, the recoded variable for encephalopathy and ventilator support shows marked differences: recipients with neither condition have the best survival, whereas survival for those on ventilator support (with or without encephalopathy) is substantially worse. Living donation yields uniformly better survival, and donor organs from younger donors also show survival benefits.

### Graft survival

Tables and figures for graft survival are provided in supporting information.

#### Model estimation

When these steps were repeated to evaluate graft survival, the same covariates were significant in univariable models, except for primary source of payment, use of partial/split organs, and cold ischemia time. We also retained the original race/ethnicity categories because the model converged without recoding. Covariates were combined for multivariable estimation, and non-significant covariates were excluded. The final graft survival model included the same covariates retained in the patient survival model (S2 Fig, S1 Table).

#### Comparison of Gray TVC and Cox PH models

Graft survival for recipients with metabolic disease and acute liver failure was similar to biliary atresia, but autoimmune disorders and the miscellaneous other category both showed increasing risk of graft failure. The increased risk is observed late (after 4 years post-transplant) with autoimmune disorders but occurs early (after 6 months post-transplant) in miscellaneous other. The effect of creatinine is also different: higher creatinine levels at transplant increase the risk of graft failure, and the risk does not dissipate over time. Recipient age and race have the same effects on graft survival as they did with patient survival. Use of living donors is uniformly better and use of older donor organs increases risk for graft failure. As with patient survival, ventilator support dramatically increases risk of early graft failure, and risks associated with encephalopathy are immediate and persist over time.

#### Example: Graft survival for a prototypical recipient

The series of graft survival curves for a prototypical recipient are provided in S3, S4 and S5 Figs.

The figures show that older children experience better graft survival than younger patients; black recipients have worse graft survival than other recipients. There are slight differences based on liver disease and no variation across creatinine levels. The greatest risks for graft failure occur with encephalopathy or ventilator support, or with the use of deceased donors. Risk also increases with the age of the donor.

## Discussion

The transplant community grapples with demand for services that far exceeds donor organ availability, and more than 2,200 individuals die on the waiting list annually [24]. The simple fact that not everyone who needs a transplant can receive one drives researchers and providers alike to evaluate processes routinely and ensure effective and fair use of scarce resources [25].

Defining goals and the best use of resources is part of the conversation. In renal transplantation, researchers have advocated for shifting organ allocation priorities from the sickest to those who will benefit the most [26, 27]. Similarly in liver transplantation, policies have evolved and now integrate local preference with medical urgency when allocating donor livers. Known as Share 35 and Share 15, the policies give priority to candidates with the greatest need and those who will benefit the most, respectively, but initial preference is extended to local and regional candidates before organs are offered nationally [28].

When considering these or other potential policy changes, accurate and robust predictions of survival (with and without transplant) are needed. Decision models and risk prediction equations are increasingly important for anticipating the impact of potential changes a priori. The current paper demonstrates advantages of time-varying approaches for estimating risk of death/graft failure in children after transplant. Overall survival was related to recipient characteristics (age, race, liver disease), severity of illness (creatinine, encephalopathy, ventilator support), and donor attributes (age, living vs. deceased donor). Beyond their importance in constructing post-transplant outcome models, these findings identify opportunities to improve pre-transplant management and highlight the challenges associated with outcome disparities related to age and race. Pre-transplant management that includes judicious use of blood products and colloid infusions and avoidance of nephrotoxic medications in settings of acute or acute-on-chronic liver failure may impact the frequency of ventilator support and renal insufficiency [29]. Racial disparities, also identified in pediatric intestinal failure and transplantation, are likely a result of myriad factors including access to care, insufficient insurance, geographic disparities, and cultural differences that require a broader collaborative to address [30].

The Gray’s TVC model provided insights that were, in some cases, surprising. Though we might expect ventilator support and creatinine levels to have waning effects on survival after the initial peri-transplant period due to survivor bias (similarities between patients who survive short-term exacerbations and those who never had problems at all), it is surprising that the effect of encephalopathy, another measure of illness severity, persists for much longer. Cross-sectional neurocognitive outcomes among children with acute liver failure identified lasting impairment of motor skills, attention, and executive function associated with clinical encephalopathy [31]. The impact of these deficits upon compliance with medications, and office and laboratory visits that may contribute to poor post-transplant outcomes requires further study. Similarly, long-term survival advantages of living donors (versus deceased donors) and non-black recipients (versus black recipients) were significant and suggest key areas for future study.

The same covariates matter in graft survival. Using older donor organs appears to increase the risk of graft failure later, but many other putative donor characteristics were not significant.

Once adjusting for these covariates, structural characteristics of the transplantation network mattered less than historically believed. Specifically, differences in organ procurement (local vs. regional sources) and cold ischemia time did not affect graft survival significantly, somewhat surprising given previous findings. Year of transplant was not significant either, suggesting that overall survival in children has been relatively stable. Where disease etiology matters (autoimmune, other chronic), the effect is not apparent in the short term but becomes important over longer periods of time. The combined effect of these covariates was illustrated through the patient prototype rather than looking at each covariate in isolation.

We encountered several limitations in our analysis, largely related to the covariates that were considered and the retrospective nature of the analysis. First, we had to exclude several covariates that had a high proportion of missingness, especially serum sodium which was not collected in OPTN data until November 2004. Data collection has expanded and these variables should be considered in the future. Second, using a stepwise procedure with backwards selection excluded several surprising variables from the final model, particularly cold ischemia time, transplant center location, and laboratory values measured at time of transplant. However, backwards selection also guards against highly correlated covariates, which may explain why variables were excluded. We therefore tested these exclusions in sensitivity analyses before finalizing the model specification. Third, our estimates relied upon retrospective information and should be validated prospectively. We also do not know yet how using more flexible prediction equations will affect the overall simulation or whether the predicted outcomes for pediatric recipients will be substantially different. Survival estimates are only one part of a larger model that needs to be considered in combination with simulation, cytokine networks and growth mixture models described previously [9], as a way to inform potential changes to allocation policies. That said, knowing that proportionality assumptions are not satisfied in this context, there is merit to using more appropriate survival models for future simulations.

The strengths of the paper include use of comprehensive, national data for pediatric liver transplant recipients, suggesting that the findings here are generalizable. One group of recipients was systematically excluded (those with a primary diagnosis of cancer) because unadjusted survival differed substantially from other diagnoses and was examined separately [18]. Using two alternative estimation methods is also a strength, demonstrating Gray’s TVC flexibility as well as its consistency with Cox when proportionality assumptions are not violated. Though the transplant community may not be surprised by the covariates found to be significant in predicting post-transplant outcomes, our results show changes in their impact over time. In this regard, there were surprising findings, such as the effect of encephalopathy, which had an effect not only during and immediately following transplantation as expected, but also remained important throughout the follow-up period. Without application of time-varying approaches, we cannot discern these and other differential effects on survival.

## Conclusion

For both patient survival and graft survival, the effect of several variables varied over time, which is consistent with our understanding of pathophysiology. However, the Cox PH model must “average” these changing effects over time, which misrepresents the impact of covariates on survival and potentially results in faulty decision making.

Our analysis demonstrated that standard Cox PH models are not appropriate statistical methods for predicting post-transplant survival. Effects of several important patient characteristics are not constant over time, and time-varying effects cannot be captured by the Cox PH model. Whatever transplant outcomes policymakers hope to optimize in the face of limited resources, good risk prediction tools are important for demonstrating the policies that will achieve those goals.

## Supporting information

S1 Fig Kaplan-Meier graph of pediatric graft survival, by liver disease category. (TIF)

S2 Fig Multivariable results for graft survival. In each graph, the effect of the covariate is graphed over time based on the Cox proportional hazards model (red line) and Gray’s time-varying model (black line, with 95% confidence intervals in gray). The model estimates can be compared to a log hazard of 0 (no effect; black dashed line).
(TIF)

S3 Fig Graft survival curves for demographic covariates of Gray’s multivariable time-varying model. In each of the graphs, the “prototypical pediatric transplant recipient” uses median values for continuous variables and modal values for categorical variables. The graft survival curve for the prototype appears in all graphs and is represented by solid circles.
(TIF)

S4 Fig Graft survival curves for clinical covariates of Gray’s multivariable time-varying model. In each of the graphs, the “prototypical pediatric transplant recipient” uses median values for continuous variables and modal values for categorical variables. The graft survival curve for the prototype appears in all graphs and is represented by solid circles.
(TIF)

S5 Fig Graft survival curves for DONOR covariates of Gray’s multivariable time-varying model. In each of the graphs, the “prototypical pediatric transplant recipient” uses median values for continuous variables and modal values for categorical variables. The graft survival curve for the prototype appears in all graphs and is represented by solid circles.
(TIF)

S1 Table Comparison of graft survival estimates (multivariable Gray TVC vs. Cox PH models). (DOCX)

## References

[1] UNOS. About: United Network for Organ Sharing; [cited 2017]. Available from: https://www.unos.org/about/.

[2] Shechter SM, Bryce CL, Alagoz O, Kreke JE, Stahl JE, Schaefer AJ, et al A clinically based discrete-event simulation of end-stage liver disease and the organ allocation process. Medical Decision Making. 2005;25(2):199–209. doi: 10.1177/0272989X04268956 15800304 10.1177/0272989X04268956

[3] SRTR. About SRTR: Scientific Registry of Transplant Recipients; [cited 2017]. Available from: https://www.srtr.org/about-srtr/mission-vision-and-values/.

[4] Squires RH Jr., Shneider BL, Bucuvalas J, Alonso E, Sokol RJ, Narkewicz MR, et al Acute liver failure in children: the first 348 patients in the pediatric acute liver failure study group. The Journal of pediatrics. 2006;148(5):652–8. Epub 2006/06/02. doi: 10.1016/j.jpeds.2005.12.051 ; PubMed Central PMCID: PMCPMC2662127. 16737880 10.1016/j.jpeds.2005.12.051 PMC2662127

[5] O'Grady JG, Alexander GJ, Hayllar KM, Williams R. Early indicators of prognosis in fulminant hepatic failure. Gastroenterology. 1989;97(2):439–45. Epub 1989/08/01. . 2490426 10.1016/0016-5085(89)90081-4

[6] Liu E, MacKenzie T, Dobyns EL, Parikh CR, Karrer FM, Narkewicz MR, et al Characterization of acute liver failure and development of a continuous risk of death staging system in children. Journal of hepatology. 2006;44(1):134–41. Epub 2005/09/20. doi: 10.1016/j.jhep.2005.06.021 . 16169116 10.1016/j.jhep.2005.06.021

[7] Lu BR, Zhang S, Narkewicz MR, Belle SH, Squires RH, Sokol RJ. Evaluation of the liver injury unit scoring system to predict survival in a multinational study of pediatric acute liver failure. The Journal of pediatrics. 2013;162(5):1010–6 e1-4. Epub 2012/12/25. doi: 10.1016/j.jpeds.2012.11.021 ; PubMed Central PMCID: PMCPMC3786160. 23260095 10.1016/j.jpeds.2012.11.021 PMC3786160

[8] Sundaram V, Shneider BL, Dhawan A, Ng VL, Im K, Belle S, et al King's College Hospital Criteria for non-acetaminophen induced acute liver failure in an international cohort of children. The Journal of pediatrics. 2013;162(2):319–23 e1. Epub 2012/08/22. doi: 10.1016/j.jpeds.2012.07.002 ; PubMed Central PMCID: PMCPMC3504621. 22906509 10.1016/j.jpeds.2012.07.002 PMC3504621

[9] Azhar N, Ziraldo C, Barclay D, Rudnick DA, Squires RH, Vodovotz Y. Analysis of serum inflammatory mediators identifies unique dynamic networks associated with death and spontaneous survival in pediatric acute liver failure. PloS one. 2013;8(11):e78202 Epub 2013/11/19. doi: 10.1371/journal.pone.0078202 ; PubMed Central PMCID: PMCPMC3823926. 24244295 10.1371/journal.pone.0078202 PMC3823926

[10] Li R, Belle SH, Horslen S, Chen LW, Zhang S, Squires RH. Clinical Course among Cases of Acute Liver Failure of Indeterminate Diagnosis. The Journal of pediatrics. 2016;171:163–70 e1-3. Epub 2016/02/03. doi: 10.1016/j.jpeds.2015.12.065 ; PubMed Central PMCID: PMCPMC4808594. 26831743 10.1016/j.jpeds.2015.12.065 PMC4808594

[11] McDiarmid SV, Anand R, Lindblad AS. Development of a pediatric end-stage liver disease score to predict poor outcome in children awaiting liver transplantation. Transplantation. 2002;74(2):173–81. Epub 2002/08/02. . 12151728 10.1097/00007890-200207270-00006

[12] Network OPaT. Policy 9: Allocation of Livers and Liver-Intestines [cited 2015]. Available from: http://optn.transplant.hrsa.gov/ContentDocuments/OPTN_Policies.pdf.

[13] Habib S, Berk B, Chang CCH, Demetris AJ, Fontes P, Dvorchik I, et al MELD and prediction of post–liver transplantation survival. Liver transplantation: official publication of the American Association for the Study of Liver Diseases and the International Liver Transplantation Society. 2006;12(3):440–7. 10.1002/lt.20721 16498643

[14] Onaca NN, Levy MF, Sanchez EQ, Chinnakotla S, Fasola CG, Thomas MJ, et al A correlation between the pretransplantation MELD score and mortality in the first two years after liver transplantation. Liver transplantation: official publication of the American Association for the Study of Liver Diseases and the International Liver Transplantation Society. 2003;9(2):117–23. 10.1053/jlts.2003.50027 12548503

[15] Brown RS Jr., Kumar KS, Russo MW, Kinkhabwala M, Rudow DL, Harren P, et al Model for end-stage liver disease and Child-Turcotte-Pugh score as predictors of pretransplantation disease severity, posttransplantation outcome, and resource utilization in United Network for Organ Sharing status 2A patients. Liver transplantation: official publication of the American Association for the Study of Liver Diseases and the International Liver Transplantation Society. 2002;8(3):278–84. Epub 2002/03/23. doi: 10.1053/jlts.2002.31340 . 11910574 10.1053/jlts.2002.31340

[16] Hayashi PH, Forman L, Steinberg T, Bak T, Wachs M, Kugelmas M, et al Model for End-Stage Liver Disease score does not predict patient or graft survival in living donor liver transplant recipients. Liver transplantation: official publication of the American Association for the Study of Liver Diseases and the International Liver Transplantation Society. 2003;9(7):737–40. Epub 2003/06/27. doi: 10.1053/jlts.2003.50122 . 12827562 10.1053/jlts.2003.50122

[17] Klein KB, Stafinski TD, Menon D. Predicting survival after liver transplantation based on pre-transplant MELD score: a systematic review of the literature. PloS one. 2013;8(12):e80661 Epub 2013/12/19. doi: 10.1371/journal.pone.0080661 ; PubMed Central PMCID: PMCPMC3861188. 24349010 10.1371/journal.pone.0080661 PMC3861188

[18] Ren Y, Chang CC, Zenarosa GL, Tomko HE, Donnell DM, Kang HJ, et al Gray's time-varying coefficients model for posttransplant survival of pediatric liver transplant recipients with a diagnosis of cancer. Computational and mathematical methods in medicine. 2013;2013:719389 Epub 2013/06/14. doi: 10.1155/2013/719389 ; PubMed Central PMCID: PMCPMC3665233. 23762197 10.1155/2013/719389 PMC3665233

[19] Roberts MS, Angus DC, Bryce CL, Valenta Z, Weissfeld L. Survival after liver transplantation in the United States: a disease‐specific analysis of the UNOS database. Liver transplantation: official publication of the American Association for the Study of Liver Diseases and the International Liver Transplantation Society. 2004;10(7):886–97. 10.1002/lt.20137 15237373

[20] Cox DR. Regression models and life-tables. Journal of the Royal Statistical Society Series B (Methodological). 1972:187–220.

[21] Gray RJ. Spline-based tests in survival analysis. Biometrics. 1994:640–52. 7981391

[22] UNOS. MELD/PELD calculator documentation: United Network for Organ Sharing; 2005 [cited 2015]. Available from: https://www.unos.org/wp-content/uploads/unos/MELD_PELD_Calculator_Documentation.pdf.

[23] UNOS. U.S. Organ Procurement and Transplantation Network Policies Rockville, MD: United Network for Organ Sharing, Richmond, VA; [cited 2017].

[24] UNOS. Data: United Network for Organ Sharing; [cited 2017]. Available from: https://www.unos.org/data/.

[25] UNOS. Policy Development: United Network for Organ Sharing; [cited 2017]. Available from: https://www.unos.org/policy/policy-development/.

[26] OPTN. The New Kidney Allocation System (KAS) Frequently Asked Questions 2014 [cited 2015]. Available from: http://optn.transplant.hrsa.gov/converge/ContentDocuments/KAS_FAQs.pdf.

[27] Israni AK, Salkowski N, Gustafson S, Snyder JJ, Friedewald JJ, Formica RN, et al New national allocation policy for deceased donor kidneys in the United States and possible effect on patient outcomes. J Am Soc Nephrol. 2014;25(8):1842–8. doi: 10.1681/ASN.2013070784 24833128 10.1681/ASN.2013070784 PMC4116061

[28] UNOS. Questions and Answers for Transplant Candidates about Liver Allocation. Richmond, VA.

[29] Lutfi R, Abulebda K, Nitu ME, Molleston JP, Bozic MA, Subbarao G. Intensive Care Management of Pediatric Acute Liver Failure. Journal of pediatric gastroenterology and nutrition. 2017;64(5):660–70. Epub 2016/10/16. doi: 10.1097/MPG.0000000000001441 . 27741059 10.1097/MPG.0000000000001441

[30] Squires RH, Balint J, Horslen S, Wales PW, Soden J, Duggan C, et al Race affects outcome among infants with intestinal failure. Journal of pediatric gastroenterology and nutrition. 2014;59(4):537–43. Epub 2014/06/12. doi: 10.1097/MPG.0000000000000456 ; PubMed Central PMCID: PMCPMC4176512. 24918984 10.1097/MPG.0000000000000456 PMC4176512

[31] Sorensen LG, Neighbors K, Zhang S, Limbers CA, Varni JW, Ng VL, et al Neuropsychological functioning and health-related quality of life: pediatric acute liver failure study group results. Journal of pediatric gastroenterology and nutrition. 2015;60(1):75–83. Epub 2014/09/25. doi: 10.1097/MPG.0000000000000575 ; PubMed Central PMCID: PMCPMC4276462. 25250681 10.1097/MPG.0000000000000575 PMC4276462

