# Supplementary figures and images for: Using time-varying models to estimate post-transplant survival in pediatric liver transplant recipients
Source: PLoS One. 2018 May 31;13(5):e0198132. doi: 10.1371/journal.pone.0198132 (PMC5978879; doi:10.1371/journal.pone.0198132)

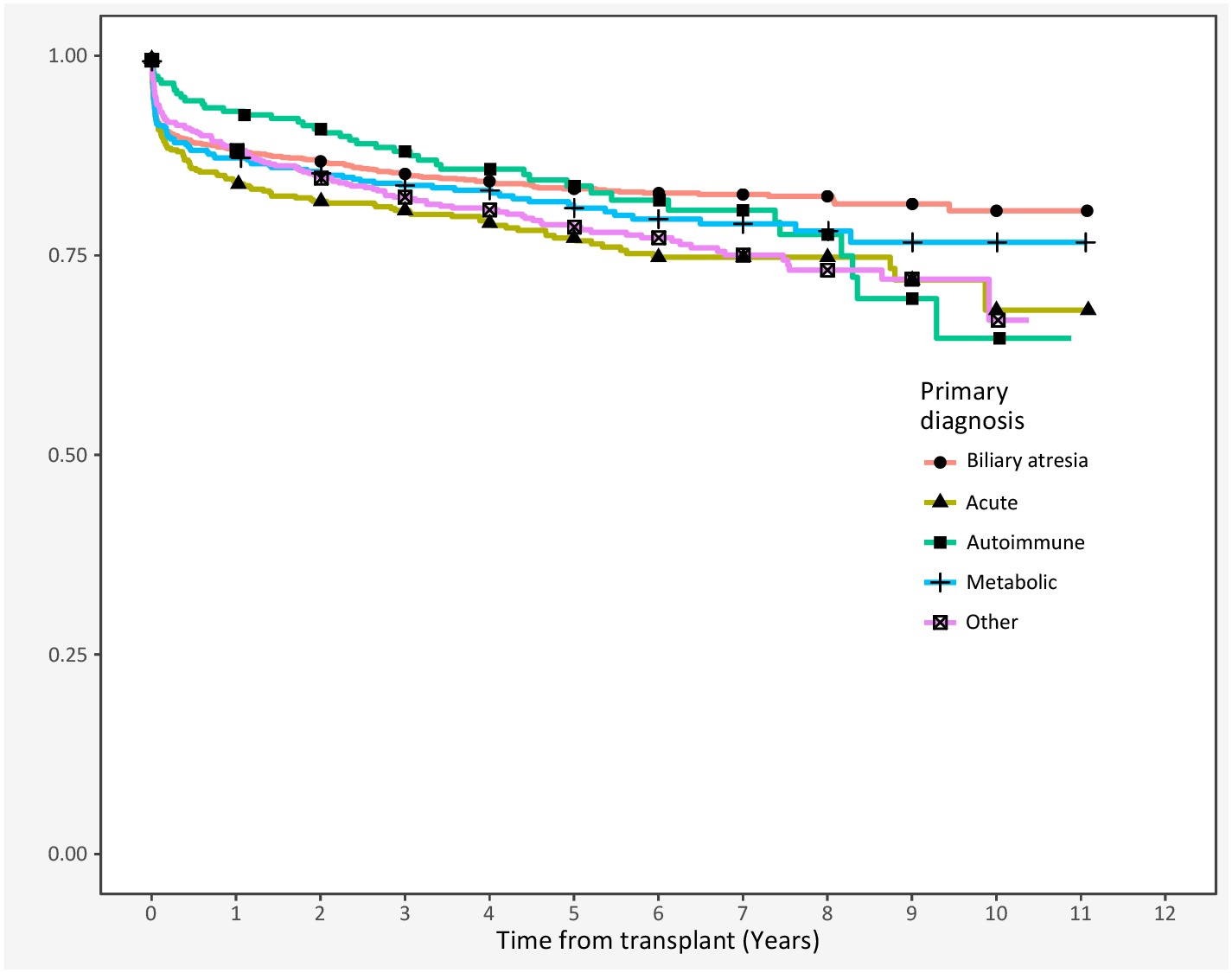

Supplement: S1 Fig — (TIF) [file pone.0198132.s001.tif]

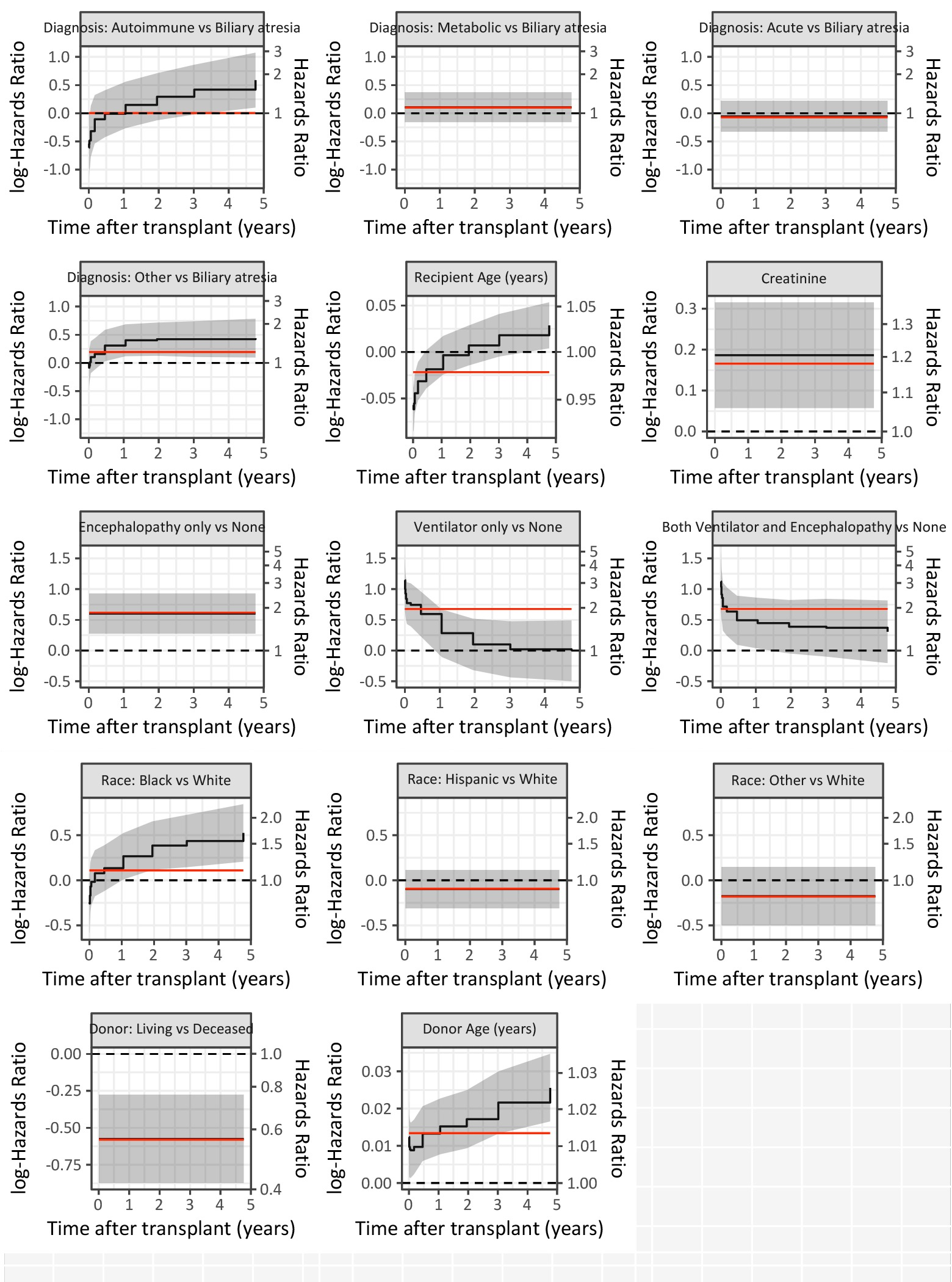

Supplement: S2 Fig — In each graph, the effect of the covariate is graphed over time based on the Cox proportional hazards model (red line) and Gray’s time-varying model (black line, with 95% confidence intervals in gray). The model estimates can be compared to a log hazard of 0 (no effect; black dashed line). (TIF) [file pone.0198132.s002.tif]

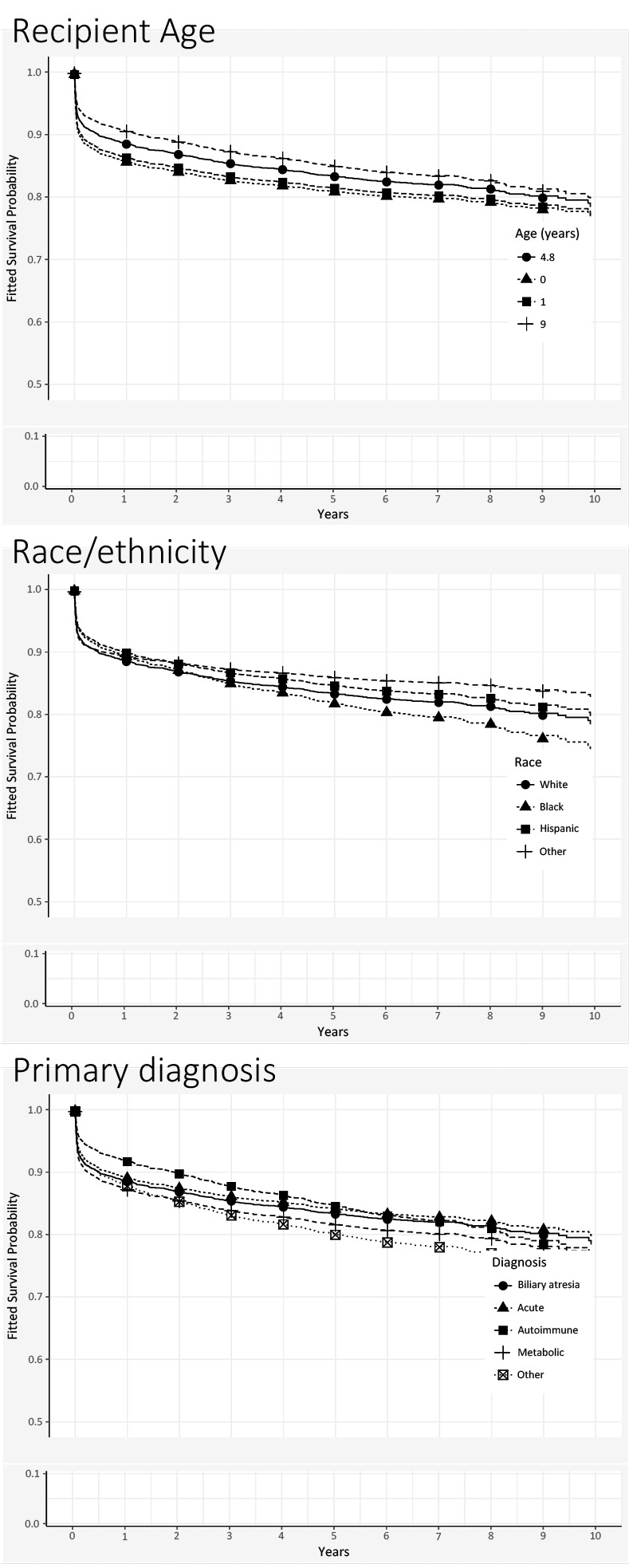

Supplement: S3 Fig — In each of the graphs, the “prototypical pediatric transplant recipient” uses median values for continuous variables and modal values for categorical variables. The graft survival curve for the prototype appears in all graphs and is represented by solid circles. (TIF) [file pone.0198132.s003.tif]

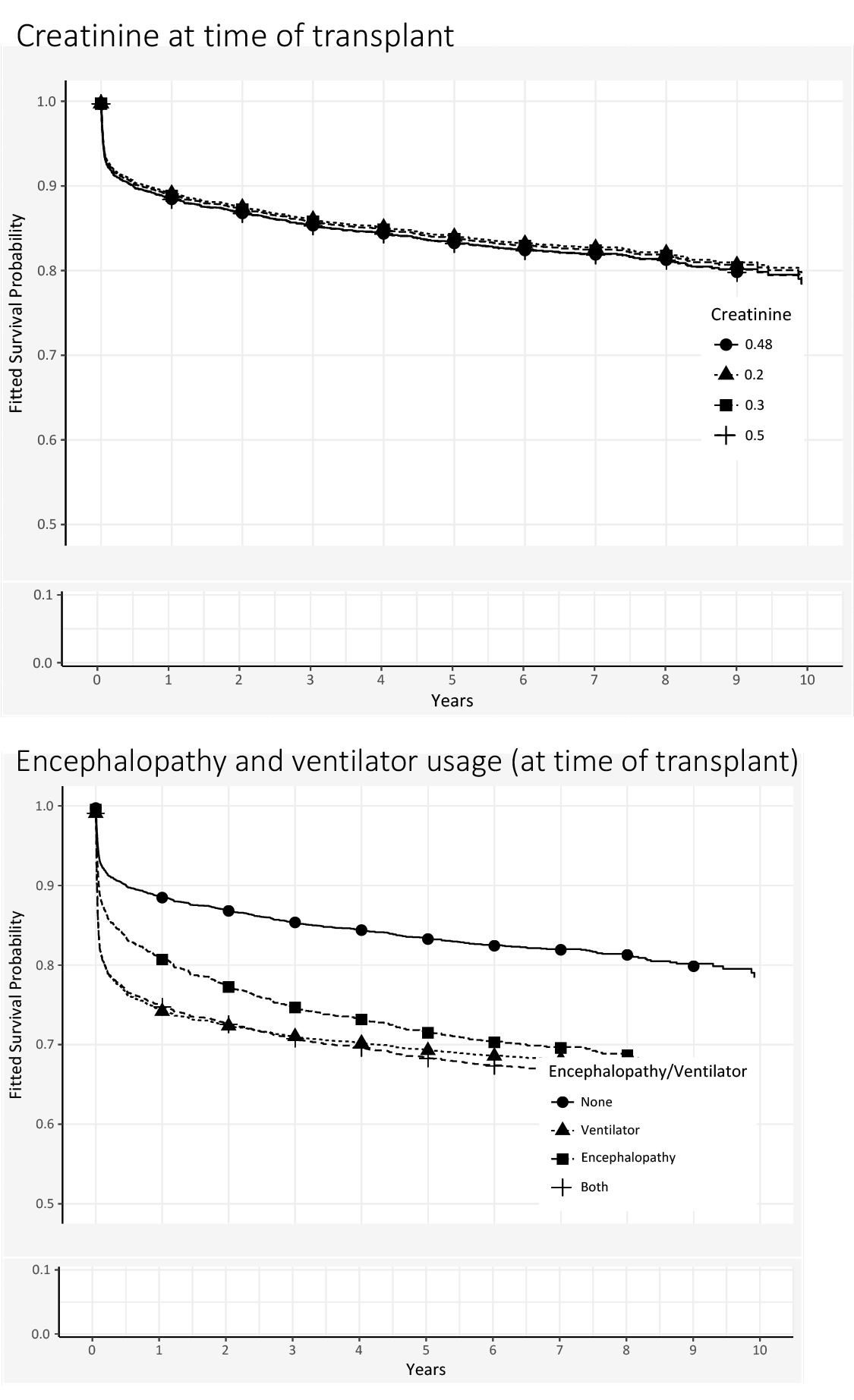

Supplement: S4 Fig — In each of the graphs, the “prototypical pediatric transplant recipient” uses median values for continuous variables and modal values for categorical variables. The graft survival curve for the prototype appears in all graphs and is represented by solid circles. (TIF) [file pone.0198132.s004.tif]

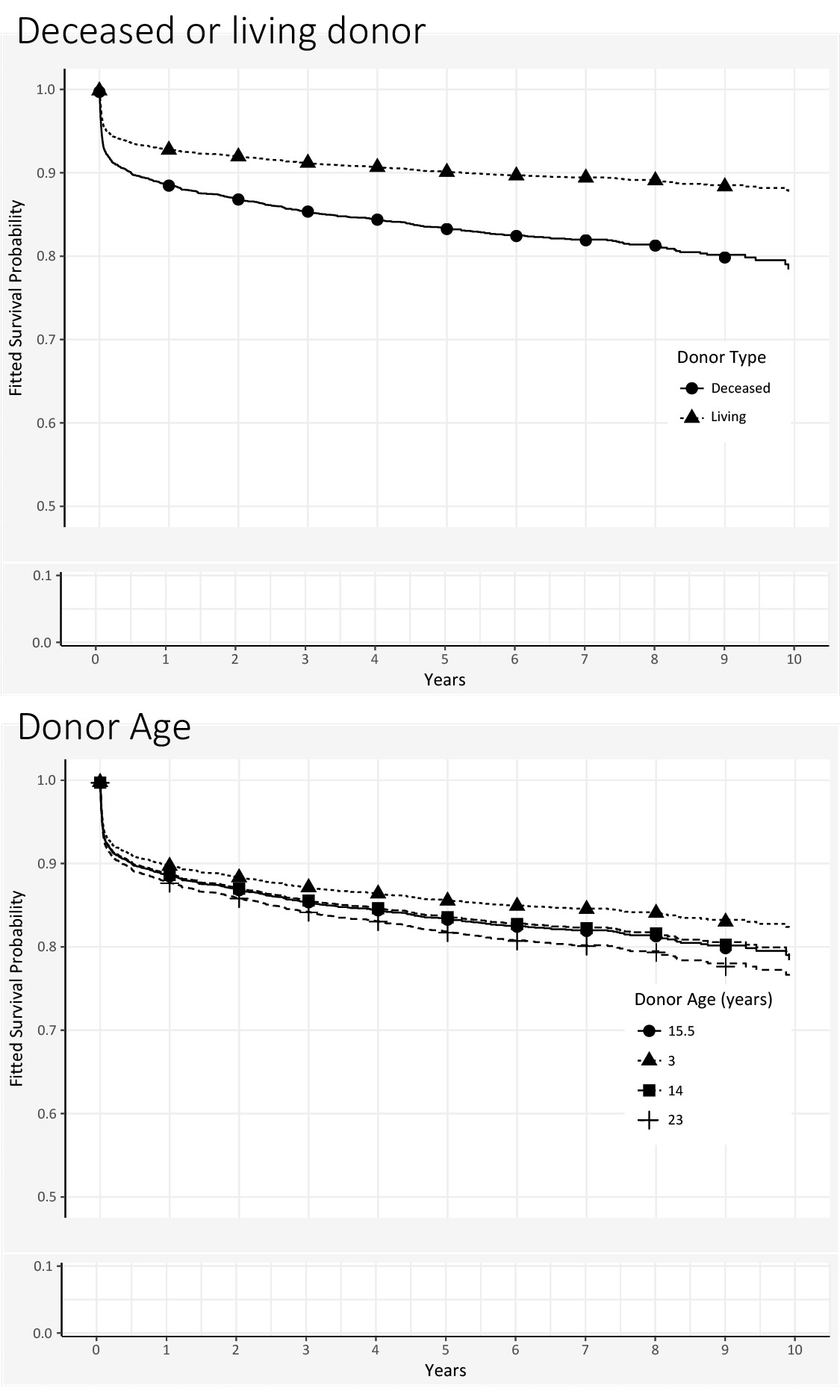

Supplement: S5 Fig — In each of the graphs, the “prototypical pediatric transplant recipient” uses median values for continuous variables and modal values for categorical variables. The graft survival curve for the prototype appears in all graphs and is represented by solid circles. (TIF) [file pone.0198132.s005.tif]
