# Supplementary material for: Using time-varying models to estimate post-transplant survival in pediatric liver transplant recipients
Source: PLoS One. 2018 May 31;13(5):e0198132. doi: 10.1371/journal.pone.0198132 (PMC5978879; doi:10.1371/journal.pone.0198132)
Supplement: S1 Table — (DOCX) [file pone.0198132.s006.docx]

Table S1. Comparison of GRAFT survival estimates (multivariable Gray TVC vs. Cox PH models).

|  |  | Gray TVC model | | |  | Cox PH model | |  | H_o_: Log hazard ratio is constant over time |
| --- | --- | --- | --- | --- | --- | --- | --- | --- | --- |
|  |  | Log hazard ratio | | H_o_: Log hazard ratio = 0 |  | Log hazard | Overall  *p* value |  |  |
| Covariate | | Min | Max |  |  |  |  |  |  |
| Recipient age | | -0.062 | 0.029 | < 0.001 |  | -0.022 | 0.018 |  | < 0.001 |
| Race/ethnicity | |  |  |  |  |  |  |  |  |
|  | White (referent) | -- | -- | -- |  | -- | -- |  | -- |
|  | Black | -0.257 | 0.525 | 0.005 |  | 0.110 | 0.320 |  | 0.001 |
|  | Hispanic | -0.311 | 0.116 | 0.371 |  | -0.094 | 0.388 |  | n/s |
|  | Other | -0.501 | 0.150 | 0.290 |  | -0.180 | 0.277 |  | n/s |
| Diagnosis | |  |  |  |  |  |  |  |  |
|  | Biliary atresia (referent) | -- | -- | -- |  | -- | -- |  | -- |
|  | Autoimmune disease | -0.611 | 0.587 | 0.021 |  | 0.003 | 0.986 |  | 0.003 |
|  | Metabolic disorder | -0.159 | 0.373 | 0.431 |  | 0.101 | 0.459 |  | n/s |
|  | Acute liver failure | -0.328 | 0.223 | 0.708 |  | -0.072 | 0.610 |  | n/s |
|  | Other chronic diagnoses | -0.084 | 0.439 | 0.019 |  | 0.192 | 0.120 |  | 0.022 |
| Serum creatinine at transplant | | 0.057 | 0.316 | 0.005 |  | 0.166 | 0.013 |  | n/s |
| Encephalopathy/mechanical ventilation at transplant | |  |  |  |  |  |  |  |  |
|  | Neither present (referent) | -- | -- | -- |  | -- | -- |  | -- |
|  | Ventilator only | -0.004 | 1.145 | < 0.001 |  | 0.677 | < 0.001 |  | < 0.001 |
|  | Encephalopathy only | 0.274 | 0.929 | < 0.001 |  | 0.619 | < 0.001 |  | n/s |
|  | Both conditions present | 0.305 | 1.124 | < 0.001 |  | 0.679 | < 0.001 |  | 0.037 |
| Use of living donor | |  |  |  |  |  |  |  |  |
|  | No (referent) | -- | -- | -- |  | -- | -- |  | -- |
|  | Yes | -0.874 | -0.274 | < 0.001 |  | -0.580 | < 0.001 |  | n/s |
| Age of donor | | 0.009 | 0.026 | < 0.001 |  | 0.013 | < 0.001 |  | 0.006 |
|  | |  |  |  |  |  |  |  |  |
